# Microsurgery Treatment as an Optimal Management of Posterior Cerebral Artery Aneurysms: A Systematic Review and Meta-Analysis

**DOI:** 10.7759/cureus.77856

**Published:** 2025-01-22

**Authors:** George Fotakopoulos, Vasiliki E Georgakopoulou, Charalabos Gatos, Grigorios Christodoulidis, Nikolaos Foroglou

**Affiliations:** 1 Department of Neurosurgery, Aristotle University of Thessaloniki, AHEPA University Hospital, Thessaloniki, GRC; 2 Department of Pathophysiology, Laiko General Hospital, Athens, GRC; 3 Department of Neurosurgery, General University Hospital of Larissa, Larissa, GRC; 4 Department of Surgery, General University Hospital of Larissa, Larissa, GRC

**Keywords:** aneurysms, microsurgery, posterior cerebral artery, posterior cerebral artery aneurysm management, posterior cerebral artery aneurysms

## Abstract

The choice of treatment of two modalities, open surgical or endovascular, in posterior cerebral artery (PCA) intracranial aneurysms must be taken based on their special characteristics. The objective of this study is to assess the potential superiority in outcomes, operative mortality, and clinical improvement after microsurgical and endovascular management repair in PCA intracranial aneurysms. Following the Preferred Reporting Items for Systematic Reviews and Meta-Analyses (PRISMA), we created this study, performing a systematic investigation on the PubMed database, with the last search carried out on June 12, 2016. The eligibility limitations were that only full text was used in the English language, and double-checking was applied. Extracted data was organized on a standard table form, including first author, publication year, general number of patients and patients at follow-up, mortality rate (with 30 days from the selecting treatment), improvement (showing postoperatively at the clinical progress (muscle strength, thinking ability, and disorientation, due to ischemic infarctions following parent vessel occlusion) for the patients of both modalities. There were eight articles that matched our study criteria. The total study population included 8,863 patients with an aneurysm, 184 (2.07%) of which had an aneurysm at the different segments of the PCA. The pooled results revealed no statistically significant difference between the two groups, in terms of mortality, but with substantial statistical results concerning clinical improvement. We concluded that the aneurysmal site and size do not influence the treatment outcome. However, clinical improvement was a statistically significant factor, demonstrating the superiority of open surgical management over endovascular treatment (EVT) for PCA aneurysms. The selection of the appropriate procedure for every case must be done based on its special characteristics.

## Introduction and background

Posterior cerebral artery (PCA) aneurysms are rare, comprising only 1%-2% of all intracranial aneurysms [1,2]. Due to their low incidence, PCA aneurysms, one of several types of posterior circulation aneurysms, are often studied in small sample sizes, with only a few institutional studies reporting more than 10 patients [1,3-33]. Furthermore, the majority of these studies report fewer than 25 cases [1,4,6-15,18,20,21,23,24,26-28,30-33].

Microsurgical treatment for posterior circulation aneurysms is currently considered a viable alternative to endovascular therapy, particularly in cases where endovascular treatment (EVT) is either unsuitable or unsuccessful [34]. However, this generalized approach does not fully capture the complexity of PCA aneurysms, which differ in some respects from aneurysms located at bifurcations or the basilar tip [10]. The deep anatomical location of PCA aneurysms, the extensive access required to reach terminal vascular segments, and their proximity to critical brain structures make their management particularly challenging [35,36].

At present, no single treatment modality consistently yields optimal results. Various neurosurgical approaches have been proposed for the microsurgical management of PCA aneurysms [19,24]. Meanwhile, endovascular techniques have gained widespread adoption across neurovascular centers globally due to their minimally invasive nature and efficacy [5,21,37].

Although significant research has been conducted on the advantages and limitations of microsurgical and endovascular techniques for intracranial aneurysms in general, specific data on PCA aneurysms remain sparse [10,34,38]. The objective of this meta-analysis was to compare the outcomes of surgical and endovascular modalities in the management of PCA aneurysms, with a focus on safety and clinical efficacy, to establish a foundation for refining future treatment strategies.

## Review

Materials and methods

**Figure 1:**
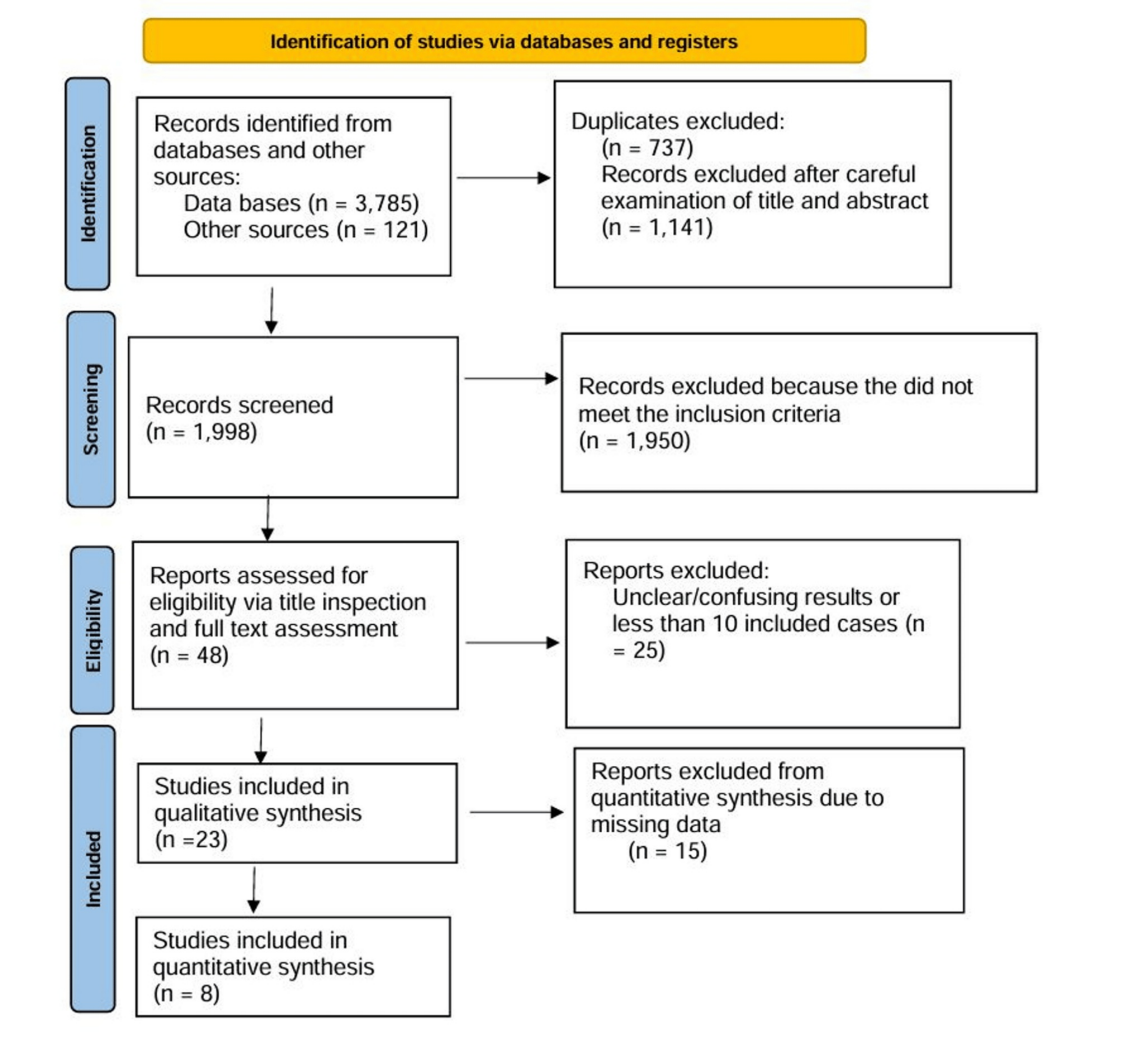
Flowchart of the study selection process

Following the Preferred Reporting Items for Systematic Reviews and Meta-Analyses (PRISMA) [39], we created this study, performing a systematic investigation on the PubMed database independently by two investigators focused on titles and abstracts. Where required, a third author assessed the selection. The keywords were "posterior cerebral artery," "posterior cerebral artery aneurysms," and "posterior cerebral artery aneurysms management." The aim of this meta-analysis was to find studies that revealed evaluations of surgical and endovascular management for PCA intracranial aneurysms. Because of low occurrence, most of the institutional studies on PCA aneurysms are few, and only a few articles with more than 10 patients have earlier been reported [1,3-33]. The last search was carried out on June 12, 2016. The eligibility limitations were that only full text was used in the English language, and double-checking was applied. Lastly, we collected 23 articles [1,3-25] meeting the criteria. In addition to these articles, in order to be included in the final pool, we had to compare the outcomes of the two methods (surgical or endovascular). We also excluded studies that compared EVT and microsurgery simultaneously, as we aimed to include studies that focused exclusively on one of the two modalities to minimize publication bias. Finally, eight articles were recognized as suitable for our meta-analysis (Figure 1) [4,5,7,8,10,14,15,19].

The next step was to organize a standard table from all the extruded data counting on the first author with publication year, the total amount of patients, operative mortality (within 30 days from the selected management), improvement (showing postoperatively at the clinical progress (muscle strength according to the Medical Research Council (MRC), thinking ability, and disorientation, due to infarctions following parent vessel occlusion) for the patients of both modalities (Table 1).

**Table 1:** Design and baseline characteristics of the trials included in the present meta-analysis PCA: posterior cerebral artery; Surg: surgical or radiosurgical management; Endov: endovascular management; NR: not reported

Author, year (Ref.)	Total number of aneurysms	Sample size with PCA aneurysms		Mean age (years)	No of males	Location												Shape				Follow-up (years)	Mean aneurysm diameter (mm)	Improvement		Mortality	
						P1		P1/P2		P2		P2/P3		P3		P4		Saccular		Fusiform							
		Surg	Endov			Surg	Endov	Surg	Endov	Surg	Endov	Surg	Endov	Surg	Endov	Surg	Endov	Surg	Endov	Surg	Endov			Surg	Endov	Surg	Endov
Chang et al., 2010 [4]	33	9	24	44	15	NR	NR	NR	NR	NR	NR	NR	NR	NR	NR	NR	NR	4	21	5	3	NR	NR	8	17	1	2
Goehre et al., 2016 [5]	135	32	6	NR	36	16	2	10	1	4	1	0	0	2	2	0	0	23	5	9	1	1	NR	31	3	0	0
Hamada et al., 2005 [7]	1853	16	5	49.8	10	1	1	8	0	6	4	0	0	0	0	1	0	NR	NR	NR	NR	NR	14	11	5	2	1
Kocaeli et al., 2009 [8]	946	6	6	47.28	5	1	1	3	0	1	4	0	0	0	0	1	1	6	3	0	3	NR	11	5	4	0	0
Kim et al., 2013 [10]	25	15	10	52	12	6	3	3	3	5	3	1	1	0	0	0	0	NR	NR	NR	NR	43.2	NR	13	7	0	0
Suzuki et al., 2003 [14]	12	2	2	53	3	0	0	0	0	1	1	1	1	0	0	0	0	1	1	1	1	11	9.1	2	1	1	0
Park et al., 2015 [15]	5829	2	19	44.5	11	0	8	0	6	1	2	1	2	0	1	0	0	2	15	0	4	NR	16.6	2	16	1	0
Taylor et al., 2003 [19]	30	28	2	46	12	17	1	7	0	2	0	1	1	1	0	0	0	11	2	17	0	NR	NR	24	1	0	1

In order to evaluate the risk of bias in any eligible article, we used also the Cochrane Collaboration tool, the Newcastle-Ottawa Scale (NOS) (Table 2) [40].

**Table 2:** Newcastle-Ottawa Scale (NOS) quality assessment of final article pool Retro, retrospective; Prosp: prospective

Author, year (Ref.)	Study design	Newcastle-Ottawa Scale			
		Selection	Comparability	Exposure	Total scores
Chang et al., 2010 [4]	Retro	3	3	2	8
Goehre et al., 2016 [5]	Retro	3	2	2	7
Hamada et al., 2005 [7]	Retro	3	2	2	7
Kocaeli et al., 2009 [8]	Prosp	3	3	3	9
Kim et al., 2013 [10]	Retro	3	2	2	7
Suzuki et al., 2003 [14]	Retro	3	3	3	9
Park et al., 2015 [15]	Retro	3	3	3	9
Taylor et al., 2003 [19]	Retro	3	3	3	9

Statistical analysis was performed using suitable software (RevMan 5.3, Nordic Cochrane Centre, Cochrane Collaboration, and Copenhagen, Denmark). An estimate of included articles' odds ratio (OR) with 95% confidence interval (CI) was used, appropriate for meta-analysis and dichotomous outcomes. OR is taken as the ratio between the odds of an event in the surgical group by the odds of occurring at a similar event in the endovascular group. When the OR is <1, that favors the surgical group (and thus p < 0.05 is reflected as statistically significant). For the assessment of statistical heterogeneity (p < 0.10 or I2 > 50%), we used the Chi-square test. In addition, for the evaluation of the significant and nonsignificant heterogeneity between the eligible articles, the random-effects and fixed-effects models were applied, respectively.

Results

Firstly, there were 48 out of 3139 articles appropriate for our meta-analysis (Figure 1). After applying all exclusion criteria, 32 articles were left, for the final pool [1,3-33]. Next, ruling out articles focused only on one of the two modalities, there were eight articles left [4,5,7,8,10,14,15,19]. The total study population was 8863 patients with aneurysmal events, 184 (2.07%) of which had aneurysm at the different segments of the PCA (110 underwent surgical clipping and 74 endovascular management) (Table 1). The mean age of the patients in the studies was from 44 to 53 years, and the males were 104 (56.5%) of the 1844 cases available from eight studies (Table 1) [4,5,7,8,10,14,15,19]. All patients were divided into two groups: Group A included patients who underwent surgery for PCA aneurysms and Group B with endovascular management.

Size and Location of the PCA Aneurysm

Data for mean aneurysm diameter was given by four articles (Table 1) [7,8,14,15]. Among these studies, the mean PCA aneurysmal diameter was 10.3 mm (range: 9.1-16.6). The mean age was 40.03 years (range: 25-53), which was available from seven articles [4,7,8,10,14,15,19]. In addition, concerning PCA aneurysm location, data was extracted from eight articles [4,5,7,8,10,14,15,19]. In the P1 segment, aneurysms were located in 57 out of 184 patients (30.9%), and 41/57 (71.9%) were included in Group A and 16/57 (28.2%) were in Group B (Table 1). In P1/P2 segment, aneurysms were located in 41 out of 184 patients (22.2%), and of these, 31/41 (75.6%) were included in Group A and 10/41 (24.3%) were in Group B. Regarding the P2 segment, aneurysms were located in 35 out of 184 patients (19.0%), and these 20/35 (57.1%) were included in group A and 15/35 (42.8%) were in Group B.

Patients with Saccular PCA Aneurysm (n = 94)

Information regarding saccular aneurysms was extracted from six articles (Table 1) [4,5,8,14,15,19]. There were 94 (68.1%) out of the 138 patients with saccular aneurysms. From 94 patients, 47 of 79 (59.4%) were in Group A, and from 47 patients, 47 of 59 (79.6%) were in Group B (Table 3).

**Table 3:** Parameters for the results of the meta-analysis Surg: surgical or radiosurgical management; Endov: endovascular management; I2: the percentage of total variation across studies that is due to heterogeneity rather than chance; CI: confidence interval; mRS: modified Rankin scale

Parameter	Included trials (n)	Total patients (n)	Surgical group (n, %)	Endovascular group (n, %)	Effect estimate (OR)	95% CI	p-value	Heterogeneity (I², %)
Aneurysm shape (saccular)	6	138	79 (57.2%)	59 (42.8%)	0.54	0.13-2.23	0.40	37%
Aneurysm shape (fusiform)	6	138	32 (40.5%)	12 (20.3%)	1.85	0.45-7.64	0.40	37%
Improvement	8	184	96 (52.1%)	54 (29.3%)	3.15	1.20-8.26	0.02	3%
Mortality	5	109	5 (4.5%)	4 (3.6%)	1.16	0.13-10.61	0.89	59%
Mass effect resolution	6	140	80 (57.1%)	40 (28.6%)	2.56	1.15-5.69	0.02	18%
mRS ≤ 2 at follow-up	5	109	85 (78.0%)	70 (64.2%)	1.85	1.05-3.27	0.03	20%

Patients with Fusiform PCA Aneurysm (n = 94)

Concerning fusiform aneurysms, information was extracted from six articles (Table 1) [4,5,8,14,15,19]. There were 44 (31.8%) out of the 138 patients with fusiform aneurysms. From 44 patients, 32 of 79 (40.5%) were in Group A, and from 47 patients, 12 of 59 (20.3%) were in Group B (Table 3).

Improvement of PCA Aneurysm

**Figure 2:**
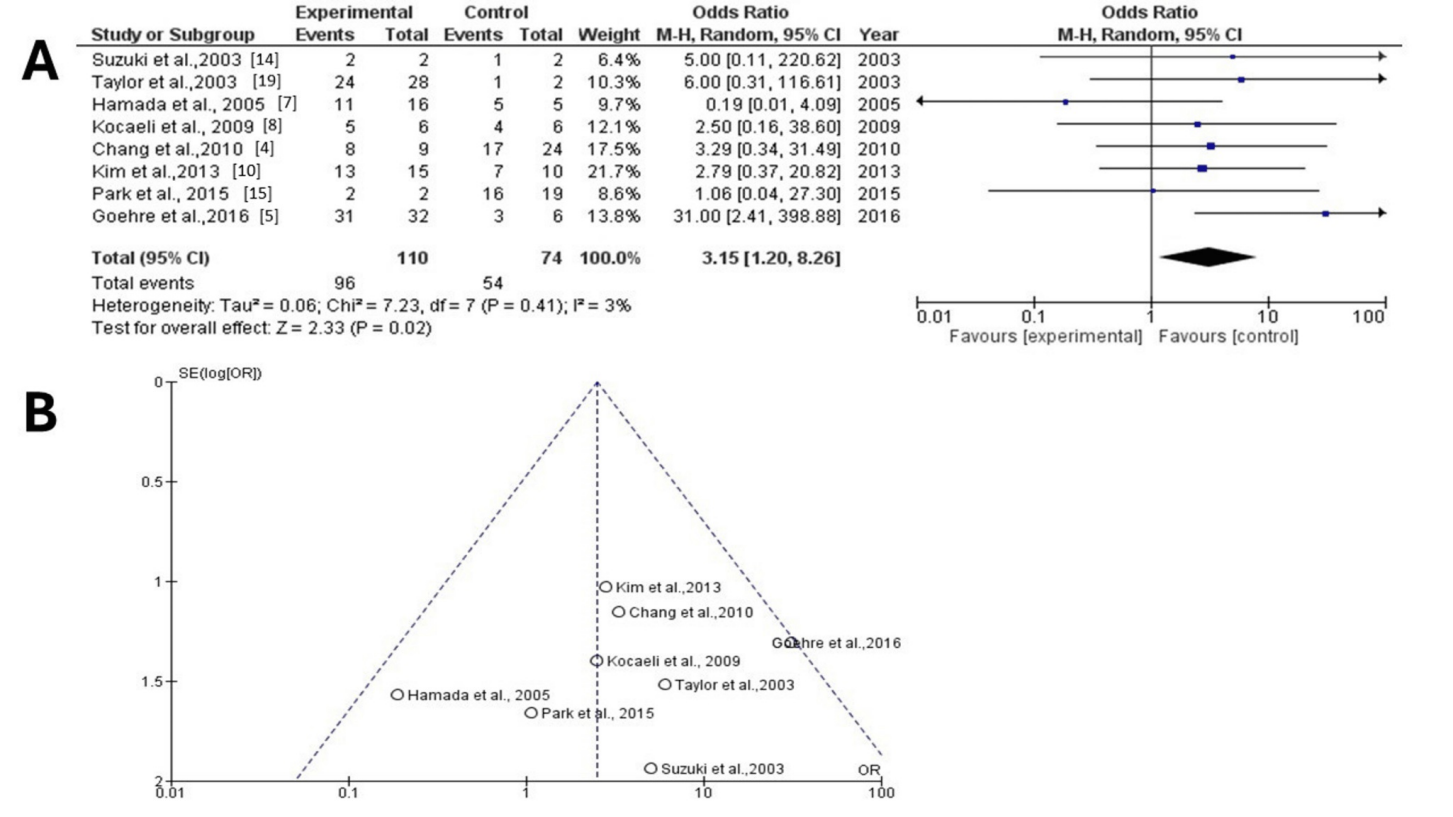
Forest and funnel plot for improvement I2: percentage of total variation across studies that is due to heterogeneity rather than chance; CI: confidence interval (A) Forest plot for improvement, with results demonstrating a statistically significant difference between groups (OR: 3.15; 95% CI: 1.20-8.26; p = 0.02), without heterogeneity (p = 0.41; I2 = 3%); (B) funnel plots for improvement in the two groups with no heterogeneity (p = 0.41; I2 = 3%)

Data was collected from eight articles [4,5,7,8,10,14,15,19]. In the entire group of patients, there were 184 patients (110 (57.7%) who underwent surgical clipping and 74 (42.3%) with endovascular management). Of these cases, 96 (52.1%) were included in the surgical group and 54 (29.3%) were in the endovascular group. Results demonstrated a statistically significant difference between groups (OR: 3.15; 95% CI: 1.20-8.26; p = 0.02), without heterogeneity (p = 0.41; I2 = 3%) (Figure 2A) (Table 3), and thus, surgical management was superior compared to endovascular management. When studying the funnel plot of the same parameter, it was observed that the study results showed no publication bias (Figure 2B). 

Mortality of PCA Aneurysm

**Figure 3:**
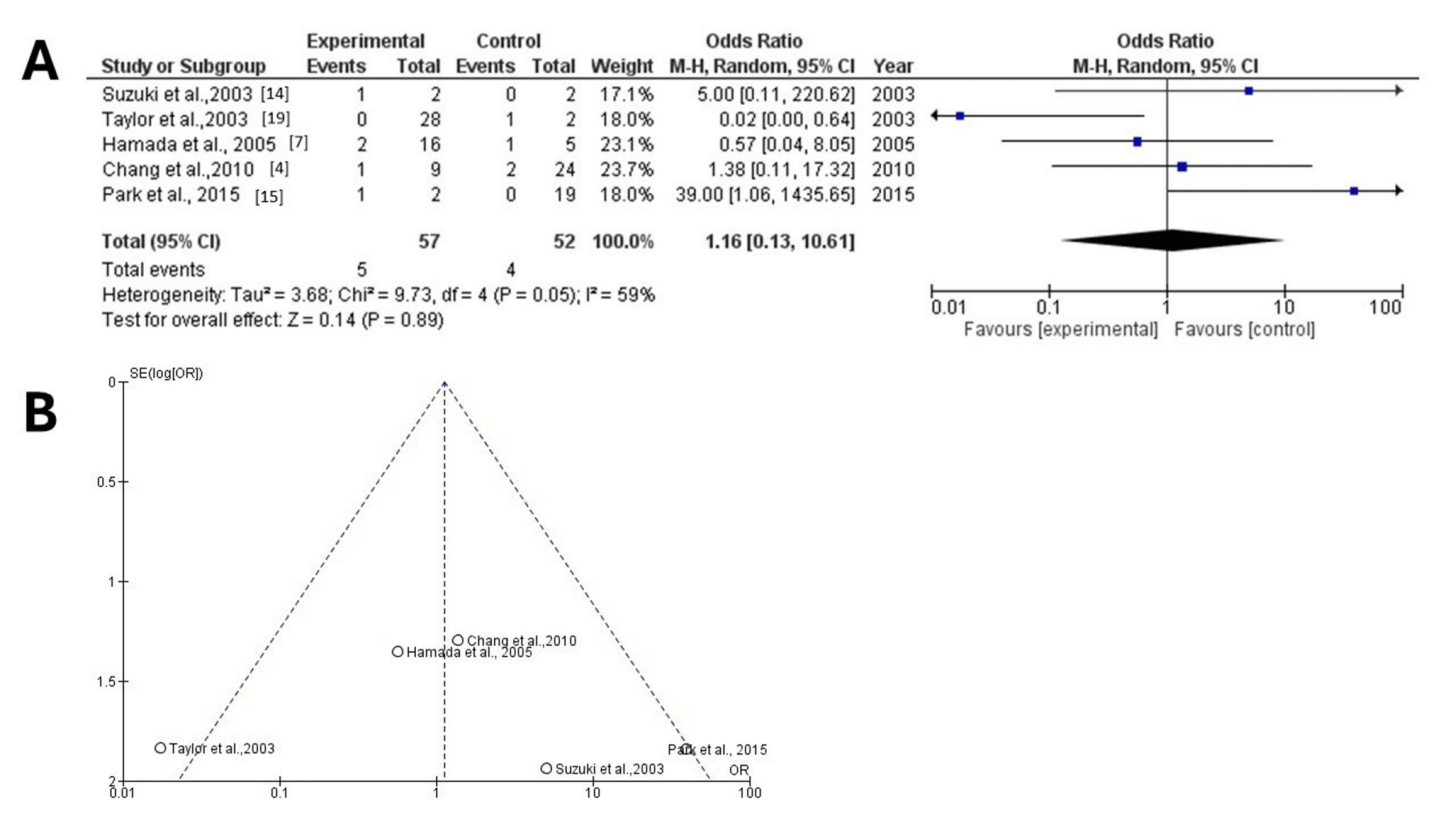
Forest and funnel plots for mortality including the study by Taylor et al. I2: percentage of total variation across studies that is due to heterogeneity rather than chance; CI: confidence interval (A) Forest plot for mortality, with results showing no statistically significant difference between the two groups (OR = 11.16; 95% CI: 0.13, 10.61; p = 0.89) but a high heterogeneity was found (p = 0.05 and I2 = 59%); (B) funnel plot for mortality in the two groups, with the study by Taylor et al.

Information regarding mortality was extracted from five articles [4,7,14,15,19] with a total of 109 patients, of whom five (4.5%) cases were in the surgical group and four (3.6%) in the endovascular one. The collective results showed no statistically significant difference between the two groups (OR = 11.16; 95% CI: 0.13-10.61; p = 0.89), but a high heterogeneity was found (p = 0.05 and I2 = 59%) (Figure 3A). Applying the "leave out one" model for the assessment of sensitivity, one article was removed at a time. After eliminating the article published by Taylor et al. in 2003 [19], which was identified as an outlier in the funnel plot due to its extreme deviation from the other studies' results, we observed improved symmetry in the plot, indicating reduced publication bias (Figure 3B) (Table 3).

**Figure 4:**
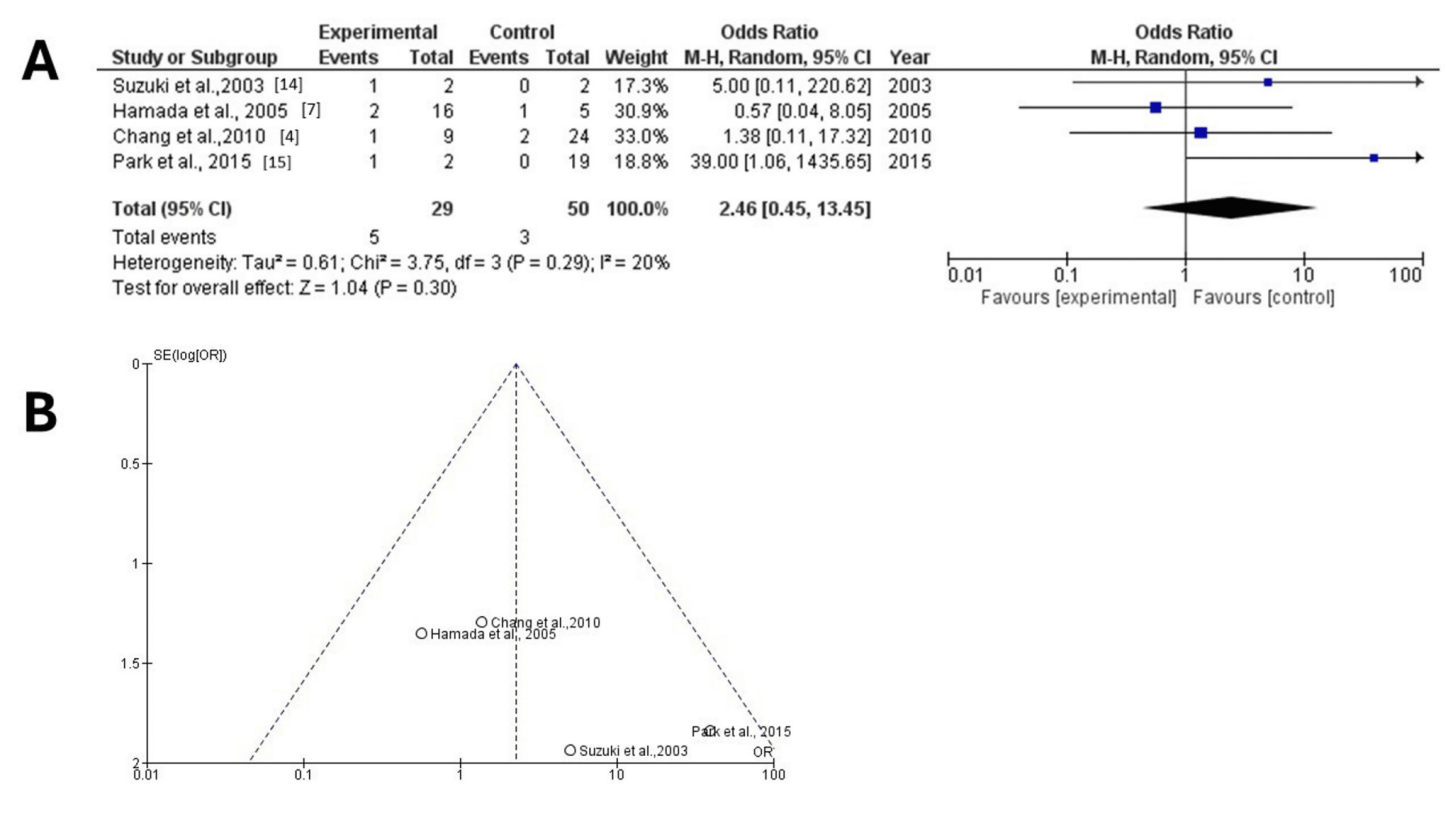
Forest and funnel plots for mortality without the study by Taylor et al. I2: percentage of total variation across studies that is due to heterogeneity rather than chance; CI: confidence interval (A) Forest plot for the same parameter without the study by Taylor et al. in 2003 demonstrated an additionally no statistically significant difference between the two groups (OR = 2.46; 95% CI: 0.45-13.45; p = 0.30) but with no heterogeneity (p = 0.29 and I2 = 20%); (B) funnel plot for mortality in the two groups, without the study by Taylor et al.

The results demonstrated an additionally no statistically significant difference between the two groups (OR =2.46; 95% CI: 0.45, 13.45; p = 0.30) (Figure 4A) but with very low heterogeneity (p = 0.29 and I2 = 20%). When assessing the funnel plot of the same variable, it was noted that the study findings without those reported by Taylor et al. in 2003 [19] showed better dispersion and no publication bias compared to the same analysis including the abovementioned study (Figure 4B).

Discussion

The current meta-analysis contains a total population of 8863 patients diagnosed with aneurysm event, and only 2.07% of them had aneurysm at the different segments of PCA. Also, the present meta-analysis suggests that surgical management of aneurysms in PCA segments is not associated with increased mortality compared to endovascular procedures. Even though PCA aneurysms concern cerebral posterior circulation and with publishing just a few institutional series with more than 10 cases, the mortality rate seems to be an independent factor of treatment modality. Interestingly, the improvement was a statistically significant factor demonstrating the advantage of surgical management of PCA aneurysms relative to the endovascular ones. These results suggest that microsurgical treatment may benefit patients with aneurysms in the PCA segments. The decision to make for the management of a PCA intracranial aneurysm is not simple and is associated with high heterogeneity, as the choice must be based on each case’s particular characteristics and the neurosurgeon’s expertise [34,38,41,42]. The reported incidence of PCA aneurysms is 1%-2%, and they are often mentioned as a simple division of posterior circulation aneurysms [1,2], creating major difficulties and very high risks of bias when performing subgroup analysis.

This very low occurrence of PCA aneurysms, with previous knowledge mostly based on a small number of cases or case reports [5], may confuse and make the decision more difficult for their treatment. We found that the PCA aneurysm incidence was 2.09% which was in agreement with the previous publications.

In literature, most PCA aneurysms were placed at the proximal PCA segments (P1 and P1/2 junction), and the mean aneurysmal diameter is around 10 mm as most of them are small (less than 7 mm) [5]. However, the deep anatomical location of PCA aneurysms, the challenging access to terminal vascular segments, and their proximity to critical brain structures make their management particularly complex and demanding [35,36].

Our meta-analysis revealed that among the studies the mean PCA aneurysmal diameter was 10.3 mm, and 53.1% of them were located in the P1 and P1/P2 junctions. PCA saccular aneurysms are easily reached by both microsurgical and endovascular methods [5]. Microsurgical management allows easy illustration control of the significant perforating branches of PCA, and the majority of complications were related to other parameters connected with the selected surgical plan [5]. EVT also has the advantage of not damaging neural structures and is reported to offer up to 70% complete aneurysmal occlusion but may need a parent vessel occlusion [32,43]. In addition, hemodynamic tension may be less possibly to incite late reopening to the parent artery. Thus, endovascular procedures could be perfect, at least for saccular PCA aneurysms [28]. We found that both endovascular and open microsurgical management for saccular PCA aneurysms are appropriate for their treatment in patient outcomes.

In the management of rare intracranial aneurysms, microsurgical techniques provide significant advantages over endovascular therapy, particularly in achieving complete aneurysm obliteration and reducing the mass effect. Microsurgery allows for direct visualization and manipulation of the aneurysm, enabling precise clipping and immediate confirmation of complete exclusion from the circulation. This approach is especially beneficial in cases of giant intracranial aneurysms, where the mass effect on adjacent neural structures can cause substantial neurological deficits. By physically removing or decompressing the aneurysm, microsurgery can more effectively alleviate these effects compared to endovascular therapy. A recent study highlighted the outcomes of microsurgical clipping versus endovascular intervention in a specific rare aneurysm subtype, demonstrating the outstanding efficacy of microsurgical techniques in achieving complete resolution and reducing mass effect. While advancements in endovascular therapy have made it a widely used option, microsurgery remains a critical tool in managing complex aneurysms, providing durable outcomes and immediate relief of symptoms caused by mass effects [44].

Concerning treatment choices for fusiform PCA aneurysms, it has been reported that as the perforating branches have their origin from the proximity of the involved segment, surgical treatment is not the first choice and an endovascular management with a balloon occlusion test can be useful, in spite of the hazards [4,5,45,46]. Overall, our study didn’t find any prevalence between endovascular and open surgery modalities for PCA aneurysm treatment.

The most serious and outcome-affecting complication for the treatment of PCA aneurysms related to the strategies selected plan is the incidence of ischemic infarctions subsequent to parent vessel occlusion [3,32]. However, the prosperous anatomical collaterals in the parent artery region make the PCA area resistant to ischaemic events [28]. The reported rate of ischemic events subsequent to parent vessel occlusion emerges to be much greater than in other aneurysm locations from the anterior and posterior circulation [3,32,47,48]. In our meta-analysis, we found that the improvement among the two modalities (surgical and endovascular) was statistically significant, showing the superiority of open surgical treatment. However, the mortality rate was the same in both choosing treatment strategies. These may be explained by the reason that the parent artery occlusion which succeeded mainly with the clipping procedure is a safe treatment option for distal PCA aneurysms, and its conservation is important for a better outcome. On the other hand, because many PCA aneurysms are fusiform or giant, entrapping is needed, as the clipping procedure is unfeasible. This increases the risk of enlarging the infarction, and the visualization of the perforating vessels may be helpful before a decision can be made regarding the final clip placement [7].

Limitations

The main limitation of the current meta-analysis was the heterogeneity among the surgical approaches discussed in the reviewed articles. In addition, the nature of complications between ruptured or unruptured aneurysms could not imprint with the accuracy of meta-analysis results, as in most of the included studies, the identification of cases concerning ruptured or unruptured aneurysms was confused. Another limitation was that as PCA aneurysms are often associated with multiple aneurysms or AVMs, in our meta-analysis, this information was not able to be extracted from all included articles, and in most series, the results were confused.

## Conclusions

We concluded that neither the aneurysmal site nor the size influences the treatment outcome. Even though that little series for PCA aneurysms has been published, the mortality rate seems to be an independent factor of the management option. In addition, the clinical improvement was a statistically significant factor, indicating the superiority of open surgical management of PCA aneurysms related to the endovascular ones. These results propose that microsurgical treatment may benefit patients with aneurysms in the PCA segments. The decision to make for the PCA intracranial aneurysm treatment is challenging and has a high heterogeneity, as the selection must be based on each case’s specific characteristics and the neurosurgeon’s abilities. Additional knowledge of a larger amount of aneurysms is needed to better identify the clinical performance and most favorable management of PCA aneurysms.
